# Why some size illusions affect grip aperture

**DOI:** 10.1007/s00221-020-05775-1

**Published:** 2020-03-17

**Authors:** Jeroen B. J. Smeets, Erik Kleijn, Marlijn van der Meijden, Eli Brenner

**Affiliations:** Department of Human Movement Sciences, Vrije Universiteit Amsterdam, van der Boechorststraat 9, 1081 BT Amsterdam, The Netherlands

**Keywords:** Visual illusion, Grasping, Prehension, Weber’s law, Inconsistency

## Abstract

There is extensive literature debating whether perceived size is used to guide grasping. A possible reason for not using judged size is that using judged positions might lead to more precise movements. As this argument does not hold for small objects and all studies showing an effect of the Ebbinghaus illusion on grasping used small objects, we hypothesized that size information is used for small objects but not for large ones. Using a modified diagonal illusion, we obtained an effect of about 10% on perceptual judgements, without an effect on grasping, irrespective of object size. We therefore reject our precision hypothesis. We discuss the results in the framework of grasping as moving digits to positions on an object. We conclude that the reported disagreement on the effect of illusions is because the Ebbinghaus illusion not only affects size, but—unlike most size illusions—also affects perceived positions.

## Introduction

Inspired by the two visual systems hypothesis (Goodale et al. 1991), an extensive literature has emerged on the question whether visual illusions affect our actions (Milner and Goodale 2006; Smeets and Brenner 2006; Franz and Gegenfurtner 2008). Sometimes illusions have an effect on action and sometimes they do not. Even within a single action, illusions frequently affect one aspect, leaving other aspects unaffected. For instance, using the Duncker illusion to change the apparent speed of a moving target that one is trying to intercept affects how quickly one moves to the target, but not where one aims to hit it (Smeets and Brenner 1995). Similarly, using the Ponzo illusion to change an object’s apparent size affects the way one lifts the object, but not the maximum grip aperture when reaching to grasp it (Brenner and Smeets 1996; Jackson and Shaw 2000). We have interpreted such findings as evidence that illusions only affect movement parameters that depend on the visual attribute that is affected by the illusion (Smeets et al. 2002). Note that this interpretation is phrased in terms of the attributes, independent of the visual cues that are used. This interpretation does not explain why a movement parameter depends on a visual attribute. For grasping, our question therefore becomes: why do participants not use the size and position of the object but two positions on its surface to control the movements of the digits?

**Fig. 1:**
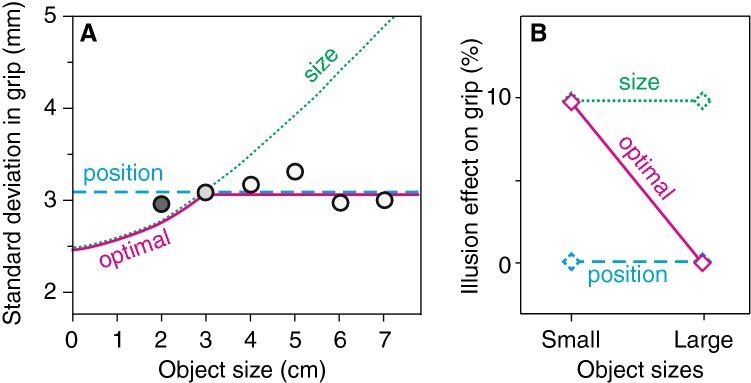
Model predictions. **a** Possible interpretation of the data (dots) of Ganel et al. (2008a), based on the calculations by Smeets and Brenner (2008). The precision in maximum grip aperture would depend differently on object size if grip aperture depended on judgments of ‘size’ (green dotted curve) than if it depended on judgments of ‘position’ (cyan dashed curve). For each object size, the ‘optimal’ attribute to rely on (purple solid curve) is the lower (most precise) of the two. **b** Prediction of the illusion effect on maximum grip aperture for small (< 3 cm) and large (> 3 cm) objects assuming a 10% effect of the illusion on perceived size

It has been proposed that we move in the way that we do to make our performance as precise as possible (Harris and Wolpert 1998), which means for many tasks that one moves in a way that minimizes the variance in the movement endpoints. Might the choice of using positions rather than size to control the digits be based on this leading to a better precision? When matching an object’s size with one’s digits, precision in reproducing the size with the distance between the digits decreases with increasing object size, whereas when moving to positions on the object’s surface, precision in the distance between the digits is independent of object size (Ganel et al. 2008a; Smeets and Brenner 2008). Smeets and Brenner (2008) provided a theoretical analysis of the measured precisions (reproduced in Fig. 1) and argued that “for objects that are larger than about 3 cm, relying on the positions of the object’s edges is more precise than relying on the object’s size”.

Based on the analysis in Fig. 1, one might argue that it would be optimal for participants to choose different information to guide the way they grasp objects of different sizes. They should guide their digits towards contact positions for large objects, but switch to scaling grip aperture to object size for objects that are smaller than 3 cm. Is there any evidence for such an optimal precision hypothesis? Unfortunately, Ganel et al. (2008a) only had one object that was smaller than 3 cm (the dark grey data point in Fig. 1). The precision of grasping that object was between the ‘position’ and the ‘optimal’ predictions. In a similar experiment, Bruno et al. (2016) did have several objects that were smaller than 3 cm and found that the variability in grip aperture increased with object size for these objects (but not for larger ones), which would be consistent with participants using judgments of size to guide their digits for the small objects. Such a trend is not visible in the data of a third study in which standard deviations in grip aperture were reported for various object sizes (Pettypiece et al. 2010), but this is not surprising given the unreliable estimates in that study, as indicated by the large scatter around the fitted lines.

Showing that the choice of attribute that is used for controlling grasping depends on object size would solve a long-standing conflict about the question whether illusions affect maximum grip aperture. The two original studies that did not find an effect of the Ponzo illusion on maximum grip aperture both used relatively large objects (6.0–7.7 cm diameter; Brenner and Smeets 1996; Jackson and Shaw 2000). More recent studies in which no effect of illusions on maximum grip aperture were found also used objects that were larger than 3 cm: the Ponzo illusion (4–4.2 cm; Ganel et al. 2008b), the diagonal illusion (5.0–9.3 cm; Stöttinger et al. 2009) and the empty space illusion (6–7 cm; Stöttinger et al. 2012). On the other hand, in experiments that involved the Ebbinghaus illusion, authors generally used target objects with a diameter of about 3.0 cm (Aglioti et al. 1995; Haffenden and Goodale 1998; Pavani et al. 1999; Franz et al. 2000; Haffenden et al. 2001; Kopiske et al. 2016). In these experiments, a consistent effect of the illusion on grip aperture is reported (Franz and Gegenfurtner 2008), although whether the effect is as large as that on perception is still under debate (Kopiske et al. 2017; Whitwell and Goodale 2017). The only illusion that seems to affect grip aperture for objects that are larger than 3 cm is the Müller–Lyer illusion (Bruno and Franz 2009). So, the optimal precision hypothesis can explain many experimental findings: size illusions generally affect maximum grip aperture if object diameter is 3 cm or smaller.

There is an alternative explanation for the experiments using the Ebbinghaus illusion showing a clear effect of perceived size on maximum grip aperture, whereas most experiments using other illusions do not. The basis of this alternative explanation is the digit-in-space hypothesis: grasping is always based on moving the tips of one’s digits to locations on the object, while approaching its surface perpendicularly, rather than shaping one’s hand opening to object size (introduced by Smeets and Brenner 1999; for a recent comprehensive review see Smeets et al. 2019). According to this hypothesis, maximum grip aperture has nothing to do with judged size, but is based on judged positions of the intended contact points. Given the finding that the Ebbinghaus illusion, unlike other illusions, influences the judged positions of points on the object’s surface (Smeets and Brenner 2019), one expects maximum grip aperture to be affected by the Ebbinghaus illusion, but not by other size illusions. This explanation does not make any distinction between large and small objects.

We therefore decided to investigate whether an illusion that does not affect perceived positions influences maximum grip aperture for objects of different sizes. We used a modified diagonal illusion, a combination of the diagonal and empty space illusions (Stöttinger and Perner 2006; Stöttinger et al. 2012), because its effect on the perceived size of a single object is similar in magnitude to that of the Ebbinghaus illusion, without any effect on the perceived positions of locations on the object’s edges (Smeets and Brenner 2019). Moreover, this illusion does not rely on surrounding objects that might influence grip aperture because they are regarded as obstacles (Haffenden et al. 2001; de Grave et al. 2005; Biegstraaten et al. 2007). The prediction of the optimal precision hypothesis is that this illusion will not affect maximum grip aperture for large objects (> 3 cm), but that the effect for small objects (< 3 cm) will be comparable to the perceptual illusion (indicated by the solid purple line in Fig. 1b). The alternative explanation based on the digit-in-space hypothesis predicts that there will be no illusion effect on maximum grip aperture, irrespective of object size (cyan dashed line in Fig. 1b). A third possibility, based on the premise that grip aperture is always based on the perceived size (Franz 2001; Kopiske et al. 2016), is that the illusion will influence maximum grip aperture as much as it does perception, irrespective of object size (green dotted line in Fig. 1b).

## Methods

### Participants

Nineteen right-handed volunteers (age range 18–60, mean 30 years) participated in the experiment. Assuming that the standard deviation in the effect of an illusion on maximum grip aperture is about 1.8 mm (Kopiske et al. 2016) and the illusion effect is about 1.5 mm (5%; Smeets and Brenner 2019) we should obtain a power of about 0.98 with 19 participants. All participants had normal or corrected-to-normal vision and were naïve with respect to the hypothesis that was tested.

### Stimuli and equipment

In choosing the illusion, we took care to ensure that the illusion-inducing context did not introduce structures that could be regarded as obstacles, that the illusion is not based on a contrast between two simultaneously presented targets, and that the effect of the illusion-inducing context was robust for small objects. To achieve this, we combined elements of two illusions: the diagonal illusion and the empty space illusion. The empty space illusion is the phenomenon that a space filled with many elements seems larger than a space that is empty or contains a single element (Luckiesh 1922; Stöttinger et al. 2012). We combined this illusion with the diagonal or Sander illusion (Luckiesh 1922; Stöttinger and Perner 2006) to produce a modified diagonal illusion. In pilot experiments, we determined parameters of the stimulus that were effective in inducing illusory length differences. The same parameters have been used in an experiment on the perceptual effects of a 2D version of this illusion (Smeets and Brenner 2019).

**Fig. 2:**
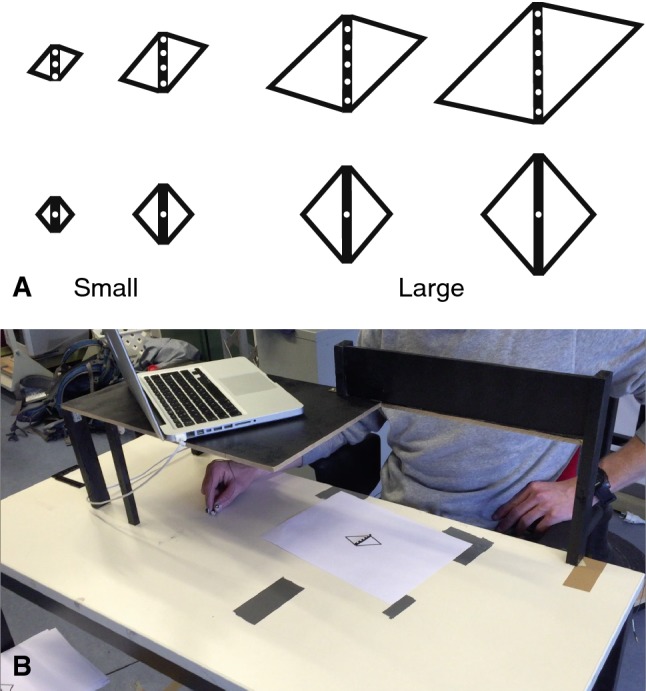
Methods. **a** The eight stimuli used in the experiment. **b** A participant in the setup with his hand holding the starting bar. The edge of the shadow of the board on which the laptop is positioned indicates about until where vision of the hand was blocked (roughly the first 25 cm of the movement)

As consistent haptic feedback is essential for normal grasping (Cuijpers et al. 2008; Schenk 2012), we let our participants grasp real 4 × 4 mm thick wooden bars. We had two short bars (1.5 and 2.5 cm) and two long bars (4 and 5 cm). We used two bar lengths within each category to ensure that participants process the visual information of the stimuli and do not simply categorize the stimuli as either ‘short’ or ‘long’ and use learned responses. For each length, we had one bar with a single white dot and another bar with several (3–5) dots. We presented each bar on a sheet of paper with a quadrilateral printed on it. There was a different sheet of paper on the table for each bar, so that the influences of the number of dots and of the kind of quadrilateral enhanced each other (Fig. 2). The bar (and thus quadrilateral) was always at the same position in front of the participant, oriented sagittally.

We instructed the participants to start their movements grasping a 40 mm long, horizontal rod, positioned 30 cm to the right of the target. When grasping this rod they had a grip aperture of 4 mm. When starting in this way, the hand’s movement is perpendicular to the bar, which not only makes grasping easy, but also ensures that the hand does not cover the illusion (Carey 2001). We deliberately chose to make the target continuously visible, because the predicted availability of feedback at contact influences how grasping movements are planned (Bozzacchi et al. 2018). Continuous vision of the target furthermore ensures direct visuomotor control without memory effects (Gentilucci et al. 1996; Westwood and Goodale 2003) and a proper visual-proprioceptive integration of location information (Smeets et al. 2006). To prevent participants from adjusting grip aperture on the basis of a direct visual comparison between target and digits (Haffenden and Goodale 1998; Franz et al. 2001; Bruno and Franz 2009), we placed a horizontal wooden board above the table, covering the first 80% of the trajectory of the hand towards the target.

The illusion works best when viewed from above, so we placed an additional vertical 10 × 40 cm wooden board in front of the participant, 20 cm above the table and 13 cm closer to the participant than the bar. This board forced participants to move their head forward to be able to see the target. In this way, we ensured that participants viewed the bar from above both when matching and grasping and that they could not see the bar between trials. We used an Optotrak 3020 (Northern Digital) to record the movements of markers attached to the nails of finger and thumb at 200 Hz. For the perceptual judgements, we placed a laptop on the wooden board, above the start location of the hand. This laptop showed a black screen (28.5 × 18 cm; surrounded by a 1 cm thick black frame) with a 2 mm thick vertical white line presented at the centre of the screen; the length of the line could be adjusted by sliding one’s finger on the trackpad.

### Tasks and procedures

To ensure that participants looked at the bar before starting to move their hand, we had participants always report the perceived size of the object directly before they grasped it. This previewing may have increased the influence that perceptual judgements of size have on the grasping movement (Glover 2002), so the influence on grasping might be overestimated.

Each trial started with the experimenter placing a stimulus: a combination of a bar and a piece of paper with a drawing of the corresponding context (Fig. 2). A line with random length was then presented on the screen of the laptop and participants moved their hand to the trackpad to match the length of the line on the screen to that of the bar. After clicking the trackpad to confirm the match, participants moved their right hand to the starting bar. They were then given a go-signal, indicating that they should pick up the bar in a single continuous movement, and place it at the target area (indicated by the thick black piece of tape on the table). This resulted in reach-to-grasp movements with a duration of about 600 ms. After placing the bar on the table, participants moved their hand back to the starting position and the next trial began.

Every stimulus was presented 10 times in a pseudorandom sequence, so in total there were 80 trials, each consisting of a judgement and a grasping movement. If the participant dropped the bar before reaching the end position, or if the bar was not grasped at the ends with a precision grip, we repeated the grasping part of the trial (this occurred in about 10% of the trials). After every 20 trials, the participants were asked whether they wanted a short break.

### Data analysis

We analysed the kinematic data of each grasping movement. We determined the grip aperture by taking the 3D distance between the markers of finger and thumb. We subsequently determined the maximum of this distance (MGA) in the part of the trajectory between movement onset and the first moment that the thumb came close to the target. The latter was defined as the first moment after the thumb had reached its peak height at which the thumb had descended to less than 1 cm above its initial height at the starting position. We chose this moment to ensure that the digits had never made contact with the object by the moment of MGA (Franz et al. 2005). We visually inspected all trials to check that this method yielded a sensible measure (e.g. that MGA was not affected by repositioning the digits after initial contact).

To determine the illusion effect, we started by taking the median response (matched length or MGA) for each participant, stimulus and task. The advantage of using the median rather than the mean is that it is robust for outliers. Moreover, it is strange to assume that MGA is normally distributed, because MGA cannot be smaller than the object size on successful grasps and it is limited by the anatomy of the participant’s hand. Therefore, the mean would be a systematically biased estimate of the central tendency of the MGA distribution. The difference between the median values for the two illusion configurations of objects of the same size provides us with four raw illusion effects for each participant for each task. To estimate the true change in size that corresponds with these raw illusion effects (Glover and Dixon 2001; Franz et al. 2005; Hesse et al. 2016), the raw illusion effects were scaled by dividing them by the slope of a fit between the eight median values for that participant (one for each combination of the two illusion configurations and four object sizes) and the corresponding actual object sizes. We subsequently expressed the illusion effects as a percentage of the mean matched length or MGA and averaged these percentages for the two small and for the two large objects. All this was done separately for each participant.

We analysed the resulting average percentage data with a repeated-measures ANOVA with factors size (small, large) and task (matching, grasping). The precision hypothesis primarily predicts an interaction, because it predicts that the illusion effect on matched length will be the same for both sizes (green dotted symbols in Fig. 1b), whereas the illusion effect on MGA will be close to zero for large objects (solid purple line). The alternative explanation based on the digit-in-space hypothesis predicts only a main effect of task, because it predicts the same illusion effect for the matching task as the precision hypothesis, combined with no illusion effect on grasping for both sizes (cyan dashed line in Fig. 1b).

In an additional analysis, we examined whether our data produce the pattern of variability within each condition that was the basis of the precision hypothesis. We tested whether objects that were perceived as being larger were matched with more variability. We also examined to what extent the variability in grip aperture depends on the grip aperture. For this, we determined the standard deviation in the maximum grip aperture and in the matching response for each participant and each of the four object sizes and two illusion configurations. We analysed both measures (variability in matched lengths and in MGA) in separate 4 (size) × 2 (illusion) repeated-measures ANOVA.

As a second additional analysis, we determined whether the illusion effects are correlated across participants. If the precision hypothesis is correct, we expect the illusion effects for MGA to be correlated with the effects for matching for the small objects, but not for the large objects. If grip aperture is always based on the perceived size, we expect the illusion effects for MGA to be correlated with the effects for matching for both object sizes. A similar correlation between the effect of the illusion on MGA for small and large objects is expected according to all hypotheses except the optimal precision hypothesis, according to which there should be no correlation. No correlation is expected if grip aperture is based on judged positions. Correlations are quite difficult to establish for this type of data because several sources of variability play a role (Franz et al. 2001). To make sure that the absence of a correlation is not due to a lack of power, we not only determined the correlations that are predicted by the various hypotheses, but also correlations that should be present irrespective of the hypothesis that is correct: that between the effects of the illusion for small and large objects.

## Results

We designed our experiment to test predictions about the effects of a size illusion on maximum grip aperture. We have three mutually excluding predictions for these effects, expressed relative to the effect of the illusion effect on the matching task (Fig. 1b). The first one is that if size is used to control grip aperture, the mean effect of the illusion on maximum grip aperture will be the same as for matching, irrespective of object size. The second one is that if positions are used to control grip aperture, the illusion will have no effect on maximum grip aperture, irrespective of object size. The third is that if the information with the highest precision is used to achieve an optimal performance, as explained in the introduction, the effect of the illusion on maximum grip aperture will be the same as for matching for small objects, but there will be no effect for large objects.

**Fig. 3:**
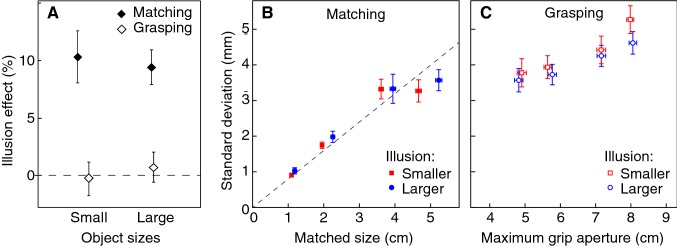
Results. All values are averages with 95% confidence intervals across participants. **a** The illusion effects for the two tasks and object size categories (small: 1.5 and 2.5 cm; large: 4.0 and 5.0 cm). **b** Within-participant standard deviation in matched size as a function of matched size for each of the four objects in the two illusion-inducing contexts. The dashed black line shows a Weber fraction of 8%. **c** Within-participant standard deviation in maximum grip aperture as a function of maximum grip aperture

The results are very clear (Fig. 3a): for both the large and the small objects, there is a considerable illusion effect for perceptual matching, and no illusion effect on maximum grip aperture. This pattern is confirmed by the ANOVA: a main effect of task (*F*_1,18_ = 88.4; *p* < 0.01) with neither an effect of size (*F*_1,18_ = 8.4 × 10^–4^; *p* = 0.98) nor an interaction (*F*_1,18_ = 2.35; *p* = 0.14). As there is no interaction between task and size (the non-significant slightly larger effect of the illusion when grasping larger objects is even in the opposite direction than predicted), we can reject our precision hypothesis (solid purple line in Fig. 1b). As the illusion effect on maximum grip aperture does not differ from zero, either for small or for large objects (see confidence intervals of the open symbols), the results are in conflict with the predictions based on the use of size and in line with the predictions based on using positions for controlling grip aperture.

The matched size increased with object size with a gain of 1.10 ± 0.02 (mean ± SEM), and increased from 1.2 cm for the 1.5 cm object to 5.0 cm for the 5 cm object. We checked whether the pattern of variability corresponds to the pattern reported by Ganel et al. (2008a). For the perceptual match (Fig. 3b), we found a strong increase in variability with object size (main effect in the ANOVA: *F*_3,54_ = 47.0, *p* < 0.001). The variability in the matched size was three times as high for the largest object than for the smallest one. There was a small effect of the illusion (*F*_1,18_ = 4.67; *p* = 0.044), with no interaction with size (*p* = 0.81). Figure 3b shows that the small effect of the illusion on the variability follows the illusion’s effect on the mean matched size: the larger appearing bars were matched with more variability than the smaller appearing ones (blue dots are to the right and above the corresponding red squares). The results can be described well with a Weber fraction of 0.08 in matching performance (dashed line), which is slightly less precise than the value of 0.06 that we used for the predictions in Fig. 1 (Ganel et al. 2008a; Smeets and Brenner 2008).

The maximum grip aperture increased in a normal way with object size (slope 0.92 ± 0.02, mean ± SEM): it increased from 4.8 cm for the 1.5 cm object to 8 cm for the 5 cm object. The within-participant standard deviation in grip aperture was on average 4.2 mm (Fig. 3c), so grip aperture was more variable than it was in the data of Ganel et al (2008a), close to the value that was reported by Bruno et al. (2016). We found a moderate increase of variability with object size (slope 0.03; *F*_3,54_ = 12.5, *p* < 0.001), without a significant effect of illusion (*p* = 0.13) or interaction (*p* = 0.68). We will come back to this finding in the discussion. The non-significant tendency for smaller appearing objects to be grasped with a more variable maximum grip aperture than larger appearing ones (red squares higher than blue circles in Fig. 3c) is in the opposite direction than the significant effect of the illusion that we found in the variability in perceptual matching (blue dots higher than red squares in Fig. 3b).

**Fig. 4:**
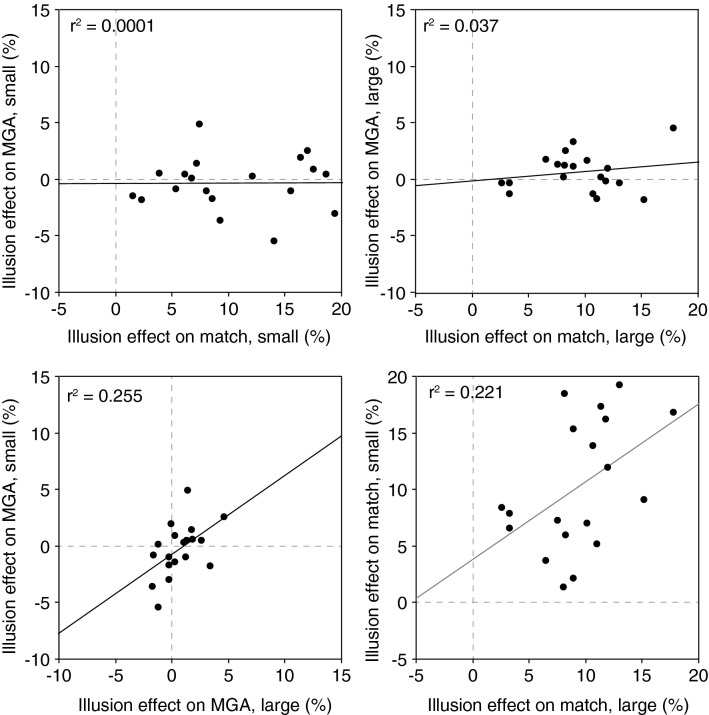
Correlations between the illusion effects across tasks and sizes. Each symbol represents the average value for a single participant. The numbers that are reported (*r*^2^) are squared Pearson’s correlation coefficients. Upper row: the illusion effect on maximum grip aperture is not correlated with the illusion effect on matching. Lower row: the illusion effect on small objects is positively correlated with the illusion effect on large objects within both tasks

We find no correlation between the illusion effects for matching and maximum grip aperture for either of the bar sizes (both *p* > 0.5; upper row in Fig. 4). This is inconsistent with any hypothesis that involves judged size being used to control grip aperture. It is consistent with the hypothesis that positions are used to control grip aperture. We find significant positive correlations between the illusion effects on maximum grip aperture for small and large objects as well as between the illusion effects on matching for small and large objects (both *p* < 0.05; lower row in Fig. 4). The latter findings indicate that the illusion effects are determined reliably enough to give our experiment the power that is needed to detect correlations within the data with this number of participants. Furthermore, the correlation that was found for grip aperture adds to the evidence against our optimal precision hypothesis.

## Discussion

The data very convincingly reject the optimal precision hypothesis: the maximum grip aperture was not affected by the illusion for any object size (open diamonds in Fig. 3a correspond to dashed cyan line in Fig. 1b). Our data also reject the hypothesis that grip aperture is based on perceived size (green dotted line in Fig. 1b): in our experiment the illusion effect on maximum grip aperture is clearly different from that on perceptual matching (solid and open symbols differ in Fig. 3a). These two conclusions are supported by the correlation analysis: the variations between participants in the (on average absent) effect on grip aperture are not correlated with the variations in effect on perceptual matching, irrespective of object size, whereas the variations are correlated between the small and large objects (Fig. 4). The results are consistent with the hypothesis that participants always use position information to guide their digits during grip formation. This suggests that they use a globally optimal strategy in grasping rather than a strategy that is optimal for the specific size of the object that is to be grasped.

### Methodological aspects

Assuming that the aim of grasping movements is to bring digits to their contact points on the object (Smeets et al. 2019), it is evident that information about such contact points is essential for a normal control of grasping movements. It is therefore not surprising that withholding information about the contact points or how they can be reached can influence the whole grasping movement. This has been demonstrated for haptic information (Cuijpers et al. 2008; Schenk 2012; Davarpanah Jazi and Heath 2016) as well as for visual information (Whitwell et al. 2016; Bozzacchi et al. 2018). We therefore designed our setup in a manner that provided our participants with both visual and haptic feedback near the point of contact, despite restricting the visual feedback to the last part of the digits’ trajectories. In this way, participants could use feedback to keep their movements calibrated, without being able to use feedback to correct them online. Not being able to directly compare grip aperture to object size during the movement is not essential for our reasoning, but it ensures that the illusion could express its full effect. Thus, both first performing the matching task and hiding the first part of the digits’ paths could result in an overestimation of the illusion’s influence on grasping with respect to naturally performing such an action.

The availability of visual information near contact is important in the study of the effect of illusions on motor control. It has been shown that the Brentano illusion has a larger effect on pointing if visual feedback is removed, which can be interpreted as a shift from relying on positions to relying on sizes (de Grave et al. 2004). The reduced effect of the illusion when vision was available was not due to online movement corrections when close to the target, because it was independent of movement speed. A possible explanation is that egocentric position information deteriorates more quickly, so it is advantageous to shift to using size when vision is removed. In grasping, a similar shift away from using positions might happen if vision of the target is blocked. This might explain why an open-loop grasping experiment in which all vision was blocked once the hand started to move found a clear effect of the diagonal illusion on maximum grip aperture (Whitwell et al. 2018).

In our experiment, the peak in grip aperture occurred when the hand was already in view. This raises the possibility that seeing the hand near the target could have led to online corrections that cancelled an illusion effect. We think this is unlikely because the hand was visible for less than 200 ms before reaching maximum grip aperture for all participants, whereas it takes more than 200 ms to complete an online correction (Oostwoud Wijdenes et al. 2011). Nevertheless, we checked whether there were clear effects of the illusion earlier in the movement by determining the effect of the illusion on grip aperture at 2/3 of the movement: when the digits were 20 cm from the starting position. At that position, the digits were still invisible. If the illusion would have influenced grip aperture in accordance with its effect on perceived size, we would expect an illusion effect of about 2 mm at that position (2/3 of the 3 mm perceptual effect). We did not find an illusion effect on the grip aperture at that position either: the mean effect is 0.3 mm, with a standard error across participants of 1.3 mm. Thus, the lack of illusion effect on maximal grip aperture is not due to online corrections based on visual feedback during the last part of the trajectory.

In addition to choices in the experimental design, we also made some choices in the data analysis that might have influenced our results. We chose to use medians rather than means for the first step of our analysis because doing so meant that we did not have to worry about outliers or about the distribution of the measurements being skewed. We repeated the analyses using means rather than medians for the first step and obviously found slight changes in the exact values, but the overall pattern of the results and of their statistical significance did not change. A second choice we made was how to scale illusion effects. We chose to use a single linear relationship between object size and performance to ensure that uncertainty about the slope (von Luxburg and Franz 2009) does not affect our conclusions. Given our hypothesis that there is difference in the way we grasp large and small objects, we could have opted for separate scaling for each object size. We therefore also repeated the analyses using this alternative way of scaling and again found a similar pattern of results.

### Interpretation of the results

Our optimal precision hypothesis was based on our interpretation of published data on the precision of grip aperture during grasping and size matching (Ganel et al. 2008a; Smeets and Brenner 2008). That study showed that the variability in size matching clearly increased with object size, whereas the variability in grip aperture did not. Looking at that data, we proposed that size may only guide grasping for small objects, because the variability in grip aperture only appeared to increase with size when grasping small objects. Our new data do not support this proposal. We confirmed that the variability increases with object size when matching the size, but we also found a modest, significant increase in variability for maximum grip aperture for the whole range object sizes (Fig. 3c). The current results, and maybe even also those of Ganel et al. (2008a), could be reconciled with Weber’s law for size by assuming that the variability in grip opening is largely masked by additional variability that is unrelated to object size. However, two experiments even report a slight decrease of variability with object size for the range of sizes that we tested (Utz et al. 2015; Bruno et al. 2016), so it is not at all certain that the variability of the maximum grip aperture increases with object size. Since the variability in grip amplitude is likely to be influenced by many factors, including purely anatomical ones, that are likely to differ between studies, we hesitate to interpret a possible small change in the variability in grip amplitude with object size. We consider the complete absence of an effect of the illusion on grip amplitude and its variability to be much more compelling.

The reported variability within participants varies considerably between studies. Our participants were about as variable in reproducing maximum grip aperture as were those of Bruno et al. (2016) and Pettypiece et al. (2010), but were much less variable than those of Utz et al. (2015). Our participants were about 30% more variable than the participants in the study of Ganel et al. (2008a), both in terms of the maximum grip aperture and in their size judgements. There are various differences between the experiments that might explain the larger variability. A first possible explanation of the 30% larger variability in our study is the larger viewing distance (more than 40 cm in our experiment versus 30 cm for Ganel et al. 2008a). This would imply that visual localisation is an important source of variability. A second possible origin of the larger variability in our experiment is that we alternated between the two tasks, whereas Ganel et al. used a blocked design. A third factor that might have played a role is the difference between the viewing conditions (Desmurget et al. 1997): Ganel’s participants could see their hand together with the target before movement onset, whereas our participants did not see their hand until it was close to the target object. As grip aperture not only depends on the present trial, but also on what happened on the previous trial (Tang et al. 2015), a fourth factor that might have influenced the variability is the range of sizes. However, as we used a smaller range than Ganel et al. (2008a), this factor would predict less variability in our study. The shape of a target is also important for the way one opens one’s hand in grasping (Verheij et al. 2012, 2014), but as both studies used thin bars, this cannot be the basis of the difference.

The strength of the illusion that we found here (about 10%) is larger than in our pencil-and-paper version of the experiment (5%; Smeets and Brenner 2019). There are several differences between the studies that might account for this difference. The main difference is the viewing geometry. In the present experiment, the bar was oriented in the sagittal direction and viewed from above. In our pencil-and-paper experiment, the bar was oriented in the fronto-parallel direction and the viewing angle was unconstrained.

A recent extensive experimental paper demonstrated convincingly that grasping is affected by the Ebbinghaus illusion and that this effect was not due to the flankers acting as obstacles (Kopiske et al. 2016). How can we reconcile their clear effect of the illusion on grip aperture (comparable in size to the perceptual effect) with the total absence of an illusion effect on maximum grip aperture in the current study? Our pencil-and-paper experiment showed that this discrepancy is actually consistent with assuming that grip aperture is based on position perception (Smeets and Brenner 1999; Smeets et al. 2019): the Ebbinghaus illusion does not only affect perceived size, but also perceived positions, whereas the modified diagonal illusion only affects perceived size.

A remaining question is why the illusions have such different effects on perceived positions while influencing perceived size in a similar manner. An explanation might be that the two illusions originate at different levels of visual processing. If so, our results suggest that the Ebbinghaus illusion affects visual processing at a stage that is common for determining size and position, whereas the modified diagonal illusion affects visual processing at a later stage. A somewhat similar distinction has been proposed to account for effects of two orientation illusions (Dyde and Milner 2002). An early origin of the Ebbinghaus illusion would also explain why the effects of the Ebbinghaus illusion on grasping and matching are correlated (Kopiske et al. 2016).

## Conclusion

The present study provides a comprehensive explanation for the apparently conflicting results that have been found when examining the effects of visual size illusions on grip aperture in reach-to-grasp movements. Size illusions themselves have no effect. The position illusion that is present in the Ebbinghaus figure is responsible for the robust effect of that ‘size’ illusion.

## Supplementary material

The data for grip aperture and perceptual settings for each trial and subject are available at https://osf.io/9cmz6/.
